# Supplementary material for: Plasma phosphorylated tau-217 correlates with brain atrophy, cognition, and cerebrospinal fluid biomarkers in a cognitively healthy community cohort
Source: Brain Commun. 2025 Oct 7;7(5):fcaf383. doi: 10.1093/braincomms/fcaf383 (PMC12550561; doi:10.1093/braincomms/fcaf383)
Supplement: fcaf383_Supplementary_Data [file fcaf383_supplementary_data.docx]

Supplementary Figures 1-5:

**Supplementary Figure 1: Added-variable plot of pTau-217 and Similarities**


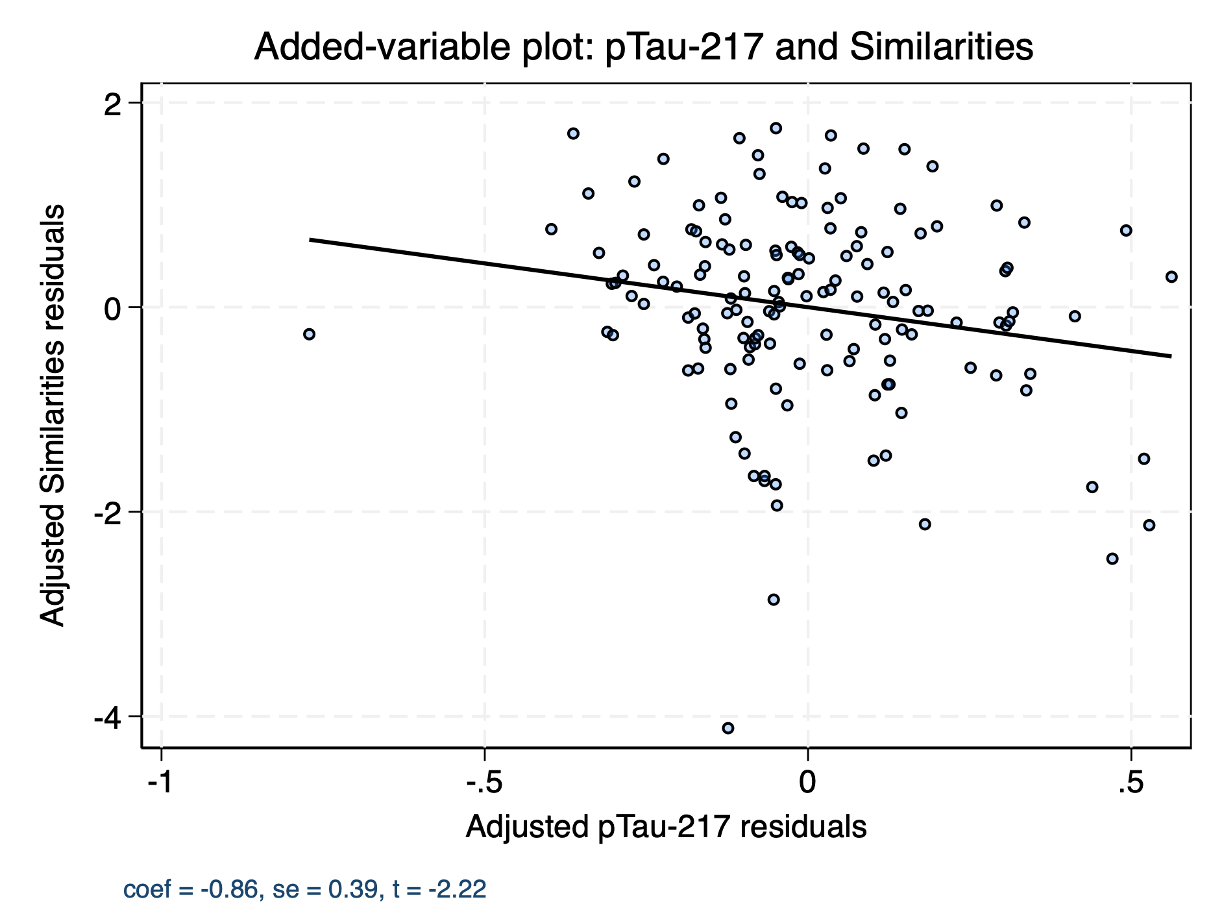


**Supplementary Figure 2: Added-variable plot of pTau-217 and MMSE**


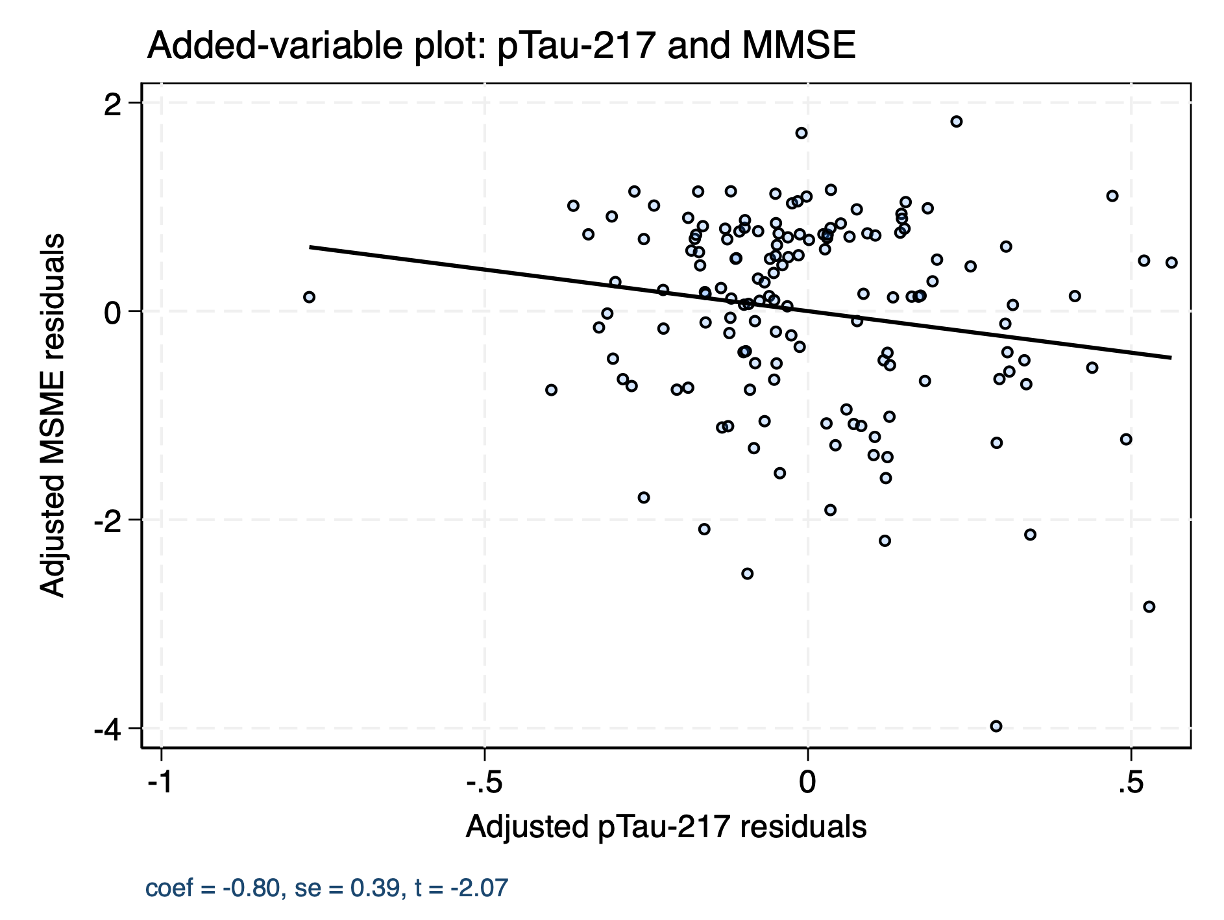


**Supplementary Figure 3: Added-variable plot of GFAP and Logical memory II (LM-II) scores**


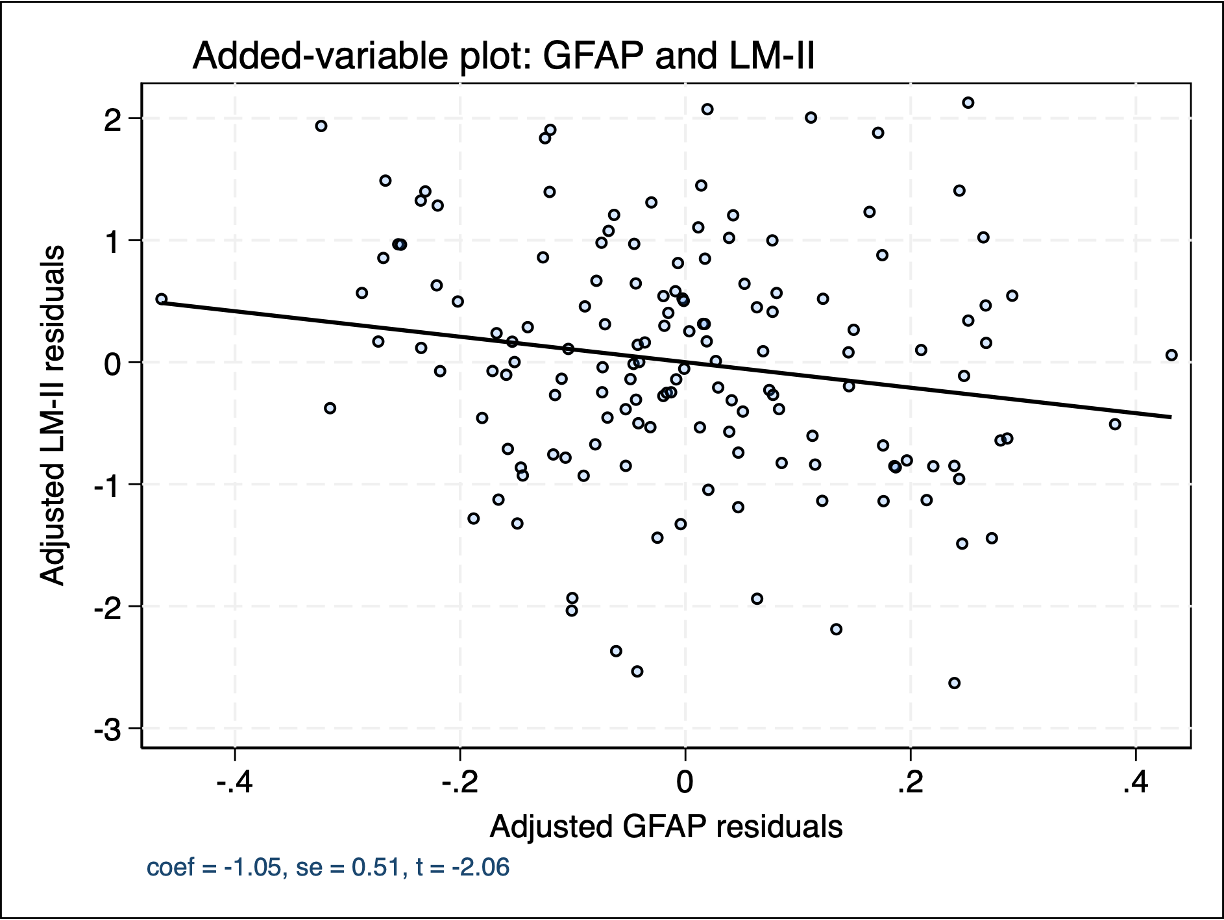


**Supplementary Figure 4: Added-variable plot of GFAP and MMSE**


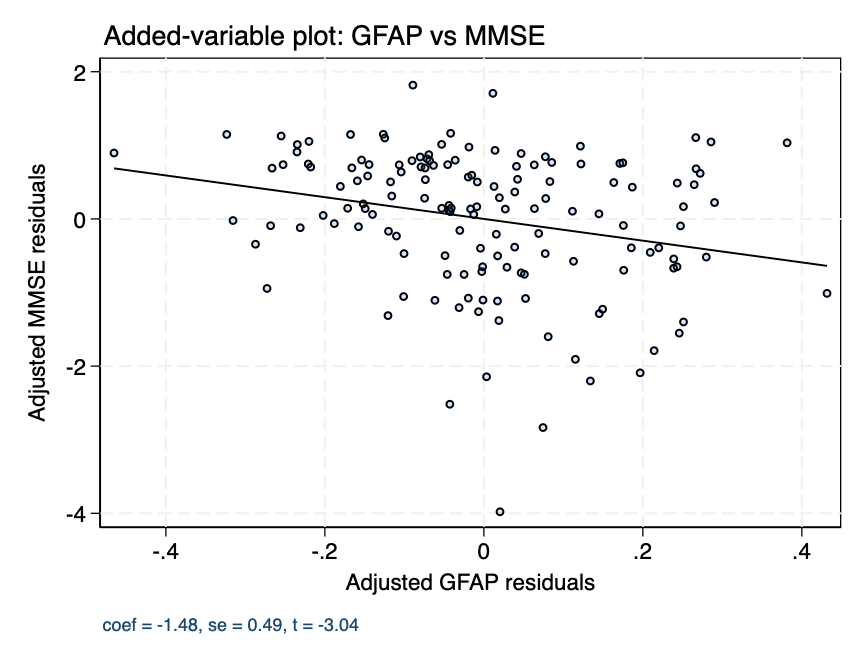


**Supplementary Figure 5: Added-variable plot of NfL and Logical memory II (LM-II) scores**


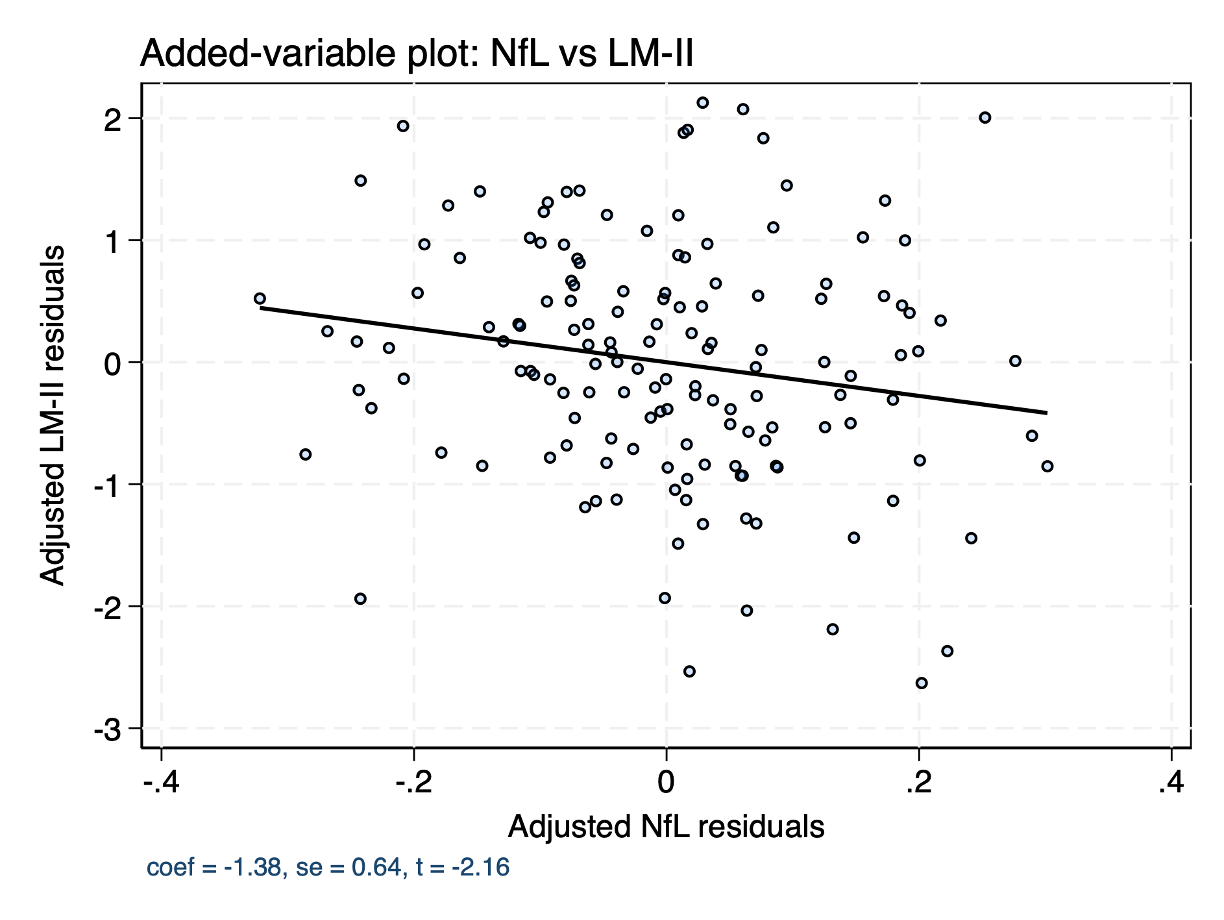


Supplementary Tables 1-5

**Supplementary Table 1: Associations of plasma biomarkers with cortical thickness on MRI, and cognitive performance (on MMSE, and tests of memory, visual processing, processing speed and verbal and abstract reasoning)**

|  | Beta | 95% C.I. | p-value |
| --- | --- | --- | --- |
| Similarities* | | | |
| pTau-217 | -0.86 | -1.62, -0.09 | 0.028 |
| pTau-181 | -0.45 | -1.55, 0.65 | 0.421 |
| GFAP | -0.54 | -1.53, 0.45 | 0.284 |
| NfL | -0.92 | -2.17, 0.33 | 0.148 |
| AB42/40 | 1.04 | -0.91, 2.98 | 0.295 |
| HVOT* | | | |
| pTau-217 | 0.12 | -0.68, 0.91 | 0.775 |
| pTau-181 | 0.24 | -0.89, 1.37 | 0.676 |
| GFAP | -0.11 | -1.13, 0.91 | 0.828 |
| NfL | -0.29 | -1.58, 0.99 | 0.653 |
| AB42/40 | -1.62 | -3.61, 0.36 | 0.108 |
| TMT-A* | | | |
| pTau-217 | 0.2 | -0.60,1.00 | 0.629 |
| pTau-181 | 0.01 | -1.13, 1.15 | 0.988 |
| GFAP | 0.76 | -1.53, 0.45 | 0.284 |
| NfL | -0.2 | -1.50, 1.10 | 0.766 |
| AB42/40 | -0.65 | -4.04 | 0.524 |
| Logical memory II* | | | |
| pTau-217 | -0.27 | -1.06, 0.52 | 0.502 |
| pTau-181 | -0.64 | -1.77, 0.48 | 0.262 |
| GFAP | -1.05 | -2.05, -0.04 | 0.041 |
| NfL | -1.38 | -2.65, -0.12 | 0.033 |
| AB42/40 | 0.12 | -1.88, 2.11 | 0.908 |
| MMSE* | | | |
| pTau-217 | -0.8 | -1.56, -0.03 | 0.041 |
| pTau-181 | -0.92 | -2.02, 0.17 | 0.096 |
| GFAP | -1.48 | -2.44, -0.52 | 0.003 |
| NfL | -0.09 | -1.34, 1.17 | 0.892 |
| AB42/40 | 1.48 | -0.45, 3.42 | 0.132 |
| Cortical thickness** | | | |
| pTau-217 | -0.1 | -0.17, -0.03 | 0.008 |
| pTau-181 | 0.01 | -0.10, 0.12 | 0.899 |
| GFAP | -0.06 | -0.15, 0.04 | 0.224 |
| NfL | -0.05 | -0.17, 0.07 | 0.434 |
| AB42/40 | 0.11 | -0.09, 0.30 | 0.278 |

*: Regression coefficients (beta) and 95% confidence intervals were obtained from linear regression adjusted for age, sex, education level, hypertension, hyperlipidemia and diabetes. Biomarker levels (expressed in pg/ml) were log-10 transformed prior to analysis. Cognitive test scores were transformed to z-scores prior to analysis. Higher cognitive test scores correspond to improved cognitive performance, except for TMT-A. Abbreviations: MMSE –Mini Mental State Examination. HVOT - Hooper Visual Organization Task. TMT-A – Trail Making Test Part-A.

**: Regression coefficients (beta) and 95% confidence intervals were obtained from linear regression adjusted for age, sex, education level, and intracranial volume. Biomarker levels (expressed in pg/ml) were log-10 transformed prior to analysis

**Supplementary Table 2: Association of plasma Aβ42 levels, cognitive performance and cortical thickness on brain MRI.**

| Outcome | Beta | 95% C.I. | p-value |
| --- | --- | --- | --- |
| MMSE * | 1.66 | 0.18, 3.14 | 0.028 |
| HVOT * | -0.37 | -1.91, 1.17 | 0.638 |
| Similarities* | 1.05 | -0.45, 2.54 | 0.169 |
| TMT-A* | -0.13 | -1.68, 1.43 | 0.871 |
| Logical memory II* | -0.34 | -1.88, 1.20 | 0.659 |
| Cortical thickness** | -0.11 | -0.03, 0.26 | 0.132 |

*: Regression coefficients (beta) and 95% confidence intervals were obtained from linear regression adjusted for age, sex, education level, hypertension, hyperlipidemia and diabetes. Biomarker levels (expressed in pg/ml) were log-10 transformed prior to analysis. Cognitive test scores were transformed to z-scores prior to analysis. Higher cognitive test scores correspond to improved cognitive performance. Abbreviations: MMSE –Mini Mental State Examination. HVOT - Hooper Visual Organization Task. TMT-A – Trail Making Test Part-A.

**Supplementary Table 3: Adjusted associations of plasma biomarkers with cortical thickness on MRI, and cognitive performance (on MMSE, and tests of memory, visual processing, processing speed and verbal and abstract reasoning)**

|  | Beta | 95% C.I. | p-value |
| --- | --- | --- | --- |
| Similarities* | | | |
| pTau-217 | -1.01 | -1.81, -0.22 | 0.013 |
| pTau-181 | -0.5 | -1.62, 0.61 | 0.376 |
| GFAP | -0.59 | -1.59, 0.41 | 0.246 |
| NfL | -0.96 | -2.22, 0.30 | 0.133 |
| AB42/40 | 1.11 | -0.85, 3.08 | 0.265 |
| HVOT* | | | |
| pTau-217 | 0.07 | -0.77, 0.90 | 0.873 |
| pTau-181 | 0.21 | -0.93, 1.36 | 0.714 |
| GFAP | -0.14 | -1.18, 0.89 | 0.782 |
| NfL | -0.32 | -1.62, 0.98 | 0.626 |
| AB42/40 | -1.6 | -3.60, 0.41 | 0.117 |
| TMT-A* | | | |
| pTau-217 | 0.11 | -0.73, 0.95 | 0.799 |
| pTau-181 | -0.05 | -1.21, 1.10 | 0.927 |
| GFAP | 0.71 | -0.32, 1.75 | 0.175 |
| NfL | -0.25 | -1.55, 1.06 | 0.71 |
| AB42/40 | -0.57 | -2.60, 1.47 | 0.582 |
| Logical memory II* | | | |
| pTau-217 | -0.39 | -1.22, 0.44 | 0.36 |
| pTau-181 | -0.71 | -1.85, 0.42 | 0.217 |
| GFAP | -1.13 | -2.14, -0.11 | 0.03 |
| NfL | -1.44 | -2.71, -0.17 | 0.027 |
| AB42/40 | 0.21 | -1.81, 2.23 | 0.839 |
| MMSE* | | | |
| pTau-217 | -0.9 | -1.70, -0.10 | 0.028 |
| pTau-181 | -0.96 | -2.06, 0.15 | 0.089 |
| GFAP | -1.52 | -2.50, -0.55 | 0.002 |
| NfL | -0.1 | -1.37, 1.17 | 0.878 |
| AB42/40 | 1.53 | -0.43, 3.48 | 0.125 |
| Cortical thickness** | | | |
| pTau-217 | -0.1 | -0.18, -0.02 | 0.01 |
| pTau-181 | 0.01 | -0.10, 0.12 | 0.859 |
| GFAP | -0.06 | -0.15, 0.04 | 0.256 |
| NfL | -0.05 | -0.17, 0.08 | 0.464 |
| AB42/40 | 0.1 | -0.09, 0.29 | 0.301 |

*: Regression coefficients (beta) and 95% confidence intervals were obtained from linear regression adjusted for age, sex, education level, APOε4 carrier status, hypertension, hyperlipidemia and diabetes. Biomarker levels (expressed in pg/ml) were log-10 transformed prior to analysis. Cognitive test scores were transformed to z-scores prior to analysis. Higher cognitive test scores correspond to improved cognitive performance, except for TMT-A. Abbreviations: MMSE –Mini Mental State Examination. HVOT - Hooper Visual Organization Task. TMT-A – Trail Making Test Part-A.

**: Regression coefficients (beta) and 95% confidence intervals were obtained from linear regression adjusted for age, sex, education level, APOε4 carrier status, and intracranial volume. Biomarker levels (expressed in pg/ml) were log-10 transformed prior to analysis.

**Supplementary Table 4: Correlation coefficients for bivariate correlations between biomarkers**

| **plasma GFAP** | **plasma NFL** | **plasma pTau-181** | **PlasmaAB42/40** | **plasma pTau-217** | **CSF GFAP** | **CSF NFL** | **CSF pTau-181** | **CSF ab42/40** | **CSF pTau-217** |  |
| --- | --- | --- | --- | --- | --- | --- | --- | --- | --- | --- |
| 1.00 | 0.43 | 0.36 | -0.28 | 0.50 | 0.67 | 0.42 | 0.31 | -0.28 | 0.37 | **plasma GFAP** |
| 0.43 | 1.00 | 0.32 | 0.06 | 0.37 | 0.39 | 0.60 | 0.17 | -0.18 | 0.31 | **Plasma NFL** |
| 0.36 | 0.32 | 1.00 | -0.29 | 0.76 | 0.36 | 0.42 | 0.60 | -0.42 | 0.57 | **Plasma pTau-181** |
| -0.28 | 0.06 | -0.29 | 1.00 | -0.36 | -0.12 | -0.04 | -0.32 | 0.50 | -0.33 | **Plasma AB42/40** |
| 0.50 | 0.37 | 0.76 | -0.36 | 1.00 | 0.36 | 0.43 | 0.66 | -0.58 | 0.76 | **Plasma pTau-217** |
| 0.67 | 0.39 | 0.36 | -0.12 | 0.36 | 1.00 | 0.67 | 0.46 | -0.22 | 0.47 | **CSF GFAP** |
| 0.42 | 0.60 | 0.42 | -0.04 | 0.43 | 0.67 | 1.00 | 0.39 | -0.23 | 0.46 | **CSF NFL** |
| 0.31 | 0.17 | 0.60 | -0.32 | 0.66 | 0.46 | 0.39 | 1.00 | -0.58 | 0.92 | **CSF pTau-181** |
| -0.28 | -0.18 | -0.42 | 0.50 | -0.58 | -0.22 | -0.23 | -0.58 | 1.00 | -0.66 | **CSF AB42/40** |
| 0.37 | 0.31 | 0.57 | -0.33 | 0.76 | 0.47 | 0.46 | 0.92 | -0.66 | 1.00 | **CSF pTau-217** |

legend: biomarker values were subject to log10-transformation prior to analysis.

**Supplementary Table 5: adjusted p-values for bivariate correlations between biomarkers**

| **plasma GFAP** | **plasma NFL** | **plasma pTau-181** | **PlasmaAB42/40** | **plasma pTau-217** | **CSF GFAP** | **CSF NFL** | **CSF pTau-181** | **CSF ab42/40** | **CSF pTau-217** |  |
| --- | --- | --- | --- | --- | --- | --- | --- | --- | --- | --- |
| 0.00E+00 | 5.28E-03 | 1.83E-02 | 6.54E-02 | 1.02E-03 | 1.44E-06 | 6.73E-03 | 4.05E-02 | 6.54E-02 | 1.61E-02 | **plasma GFAP** |
| 5.28E-03 | 0.00E+00 | 3.85E-02 | 7.22E-01 | 1.61E-02 | 1.24E-02 | 2.54E-05 | 2.68E-01 | 2.42E-01 | 4.24E-02 | **Plasma NFL** |
| 1.83E-02 | 3.85E-02 | 0.00E+00 | 5.77E-02 | 3.83E-09 | 1.83E-02 | 6.73E-03 | 2.54E-05 | 6.73E-03 | 8.97E-05 | **Plasma pTau-181** |
| 6.54E-02 | 7.22E-01 | 5.77E-02 | 0.00E+00 | 1.83E-02 | 4.22E-01 | 8.12E-01 | 3.91E-02 | 1.02E-03 | 3.07E-02 | **Plasma AB42/40** |
| 1.02E-03 | 1.61E-02 | 3.83E-09 | 1.83E-02 | 0.00E+00 | 1.83E-02 | 5.28E-03 | 1.60E-06 | 7.14E-05 | 3.42E-09 | **Plasma pTau-217** |
| 1.44E-06 | 1.24E-02 | 1.83E-02 | 4.22E-01 | 1.83E-02 | 0.00E+00 | 1.44E-06 | 2.81E-03 | 1.46E-01 | 2.19E-03 | **CSF GFAP** |
| 6.73E-03 | 2.54E-05 | 6.73E-03 | 8.12E-01 | 5.28E-03 | 1.44E-06 | 0.00E+00 | 1.08E-02 | 1.43E-01 | 2.71E-03 | **CSF NFL** |
| 4.05E-02 | 2.68E-01 | 2.54E-05 | 3.91E-02 | 1.60E-06 | 2.81E-03 | 1.08E-02 | 0.00E+00 | 5.40E-05 | 8.53E-19 | **CSF pTau-181** |
| 6.54E-02 | 2.42E-01 | 6.73E-03 | 1.02E-03 | 7.14E-05 | 1.46E-01 | 1.43E-01 | 5.40E-05 | 0.00E+00 | 1.86E-06 | **CSF AB42/40** |
| 1.61E-02 | 4.24E-02 | 8.97E-05 | 3.07E-02 | 3.42E-09 | 2.19E-03 | 2.71E-03 | 8.53E-19 | 1.86E-06 | 0.00E+00 | **CSF pTau-217** |

legend: biomarker values were subject to log10-transformation prior to analysis.
